# Supplementary material for: Splice-Junction-Based Mapping of Alternative Isoforms in the Human Proteome
Source: Cell Rep. Author manuscript; Available in PMC 2020 Jan 15. (PMC6961840; doi:10.1016/j.celrep.2019.11.026)
Supplement: 2 [file NIHMS1546469-supplement-2.zip › DF1/PXD010154/Heart/Heart_13_TTN_ASNEYGSVSCTATLTVTEPPK.pdf]

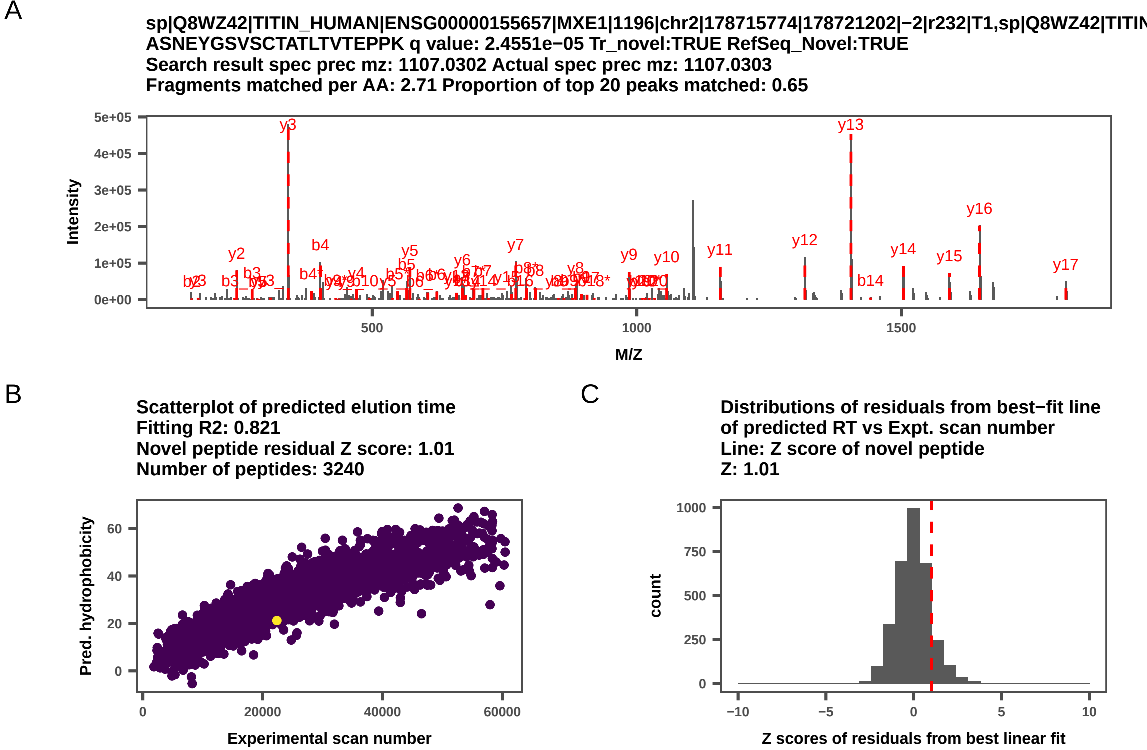
A
sp|Q8WZ42|TITIN_HUMAN|ENSG00000155657|MXE1|1196|chr2|178715774|178721202|−2|r232|T1,sp|Q8WZ42|TITIN
ASNEYGSVSCTATLTVTEPPK q value: 2.4551e−05 Tr_novel:TRUE RefSeq_Novel:TRUE
Search result spec prec mz: 1107.0302 Actual spec prec mz: 1107.0303
Fragments matched per AA: 2.71 Proportion of top 20 peaks matched: 0.65
Intensity
5e+05
4e+05
3e+05
2e+05
1e+05
0e+00
y3
y13
y16
y2
b4
y5
y7
y9
y10
y11
y12
y14
y15
y17
b14
500
1000
1500
M/Z
B
Scatterplot of predicted elution time
Fitting R2: 0.821
Novel peptide residual Z score: 1.01
Number of peptides: 3240
Pred. hydrophobicity
60
40
20
0
20000
40000
60000
Experimental scan number
C
Distributions of residuals from best−fit line
of predicted RT vs Expt. scan number
Line: Z score of novel peptide
Z: 1.01
count
1000
750
500
250
0
−10
−5
0
5
10
Z scores of residuals from best linear fit
